# Psychological flexibility and self-compassion buffer the impact of shame on depression among adults with physical disabilities

**DOI:** 10.1371/journal.pmen.0000707

**Published:** 2026-09-09

**Authors:** Michael Christopher, Xander Kahle, Kaylia Pham

**Affiliations:** 1 College of Health Sciences, Boise State University, 1910 University Drive, Boise, Idaho, United States of America; 2 School of Graduate Psychology, Pacific University, Forest Grove, Oregon, United States of America; PLOS: Public Library of Science, UNITED KINGDOM OF GREAT BRITAIN AND NORTHERN IRELAND

## Abstract

Individuals with physical disabilities experience higher rates of depression relative to the general population. Shame is a central mechanism linking disability status to poor mental health outcomes, including depression. Psychological flexibility and self-compassion have been shown to change one’s relationship to negative self-evaluations such as shame. The goal of this study was to determine if psychological flexibility and self-compassion negatively predict depression and buffer the impact of shame on depression in adults with physical disabilities. Adults with physical disabilities (*n* = 207) completed self-report measures of psychological flexibility, self-compassion, shame, and depression. Consistent with prediction, a series of multiple regressions indicated that psychological flexibility and self-compassion negatively predicted depression over and beyond shame, and they both mitigated the impact of shame on depression among adults with physical disabilities (all *p*’s < .05). These results suggest that psychological flexibility and self-compassion enhancing treatments, such as mindfulness-based interventions and acceptance and commitment therapy may be helpful in improving the mental health and wellbeing of adults with physical disabilities.

## Introduction

Individuals with physical disabilities contend with an array of chronic stressors that affect both body and mind [1,2]. While outward manifestations may be observed in functional impairments or chronic pain, the persistent strain on mental health can lead to even greater challenges [3,4]. According to the Centers for Disease Control and Prevention, people with physical disabilities are 4.6 times more likely to report frequent mental distress than the general population, with risk further heightened among those with fewer socioeconomic resources [5]. When unaddressed, this distress can progress into serious mental health conditions, including anxiety, post-traumatic stress, suicidality, and depression, all of which occur at elevated rates in this population [2–4,6]. Psychosocial factors such as stigma and shame have emerged as central mechanisms linking disability status to poor mental health outcomes [7–9].

Individuals with physical disabilities live in societies predominantly structured for those without disabilities. To engage in social systems and institutions organized around able-bodied norms, they often require accommodations merely to participate on equitable terms [10,11]. Yet inclusion under these conditions is rarely neutral, as the visibility of difference can expose individuals to subtle and overt forms of stigma [3,12]. Negative reactions from others may take the form of unsolicited staring, visible discomfort in offering assistance, or outright discrimination and harassment [12,13]. Repeated exposure to such experiences communicates that one is “other,” leading stigma to become internalized as shame [7,8,14].

Shame is a transdiagnostic emotion that arises when external devaluation becomes internalized as a sense of personal defectiveness and social undesirability [15–17]. Rooted in the perceived threat of rejection, shame motivates defensive responses such as withdrawal and isolation, which initially reduce exposure to social threat but, over time, deepen loneliness and distress [8,9,18]. Avoidance has been identified as a significant predictor of depression, anxiety, and disability among individuals with chronic pain [19]. Although research suggests a clear connection between shame and adverse mental health outcomes in people with physical disabilities, key psychological processes such as psychological flexibility and self-compassion may disrupt this pathway and serve as protective processes [7,14].

Psychological flexibility (PF) comprises six interrelated processes that enable individuals to engage with difficult internal experiences openly and adaptively while pursuing actions consistent with personal values [20,21]. PF is the central mechanism targeted in acceptance and commitment therapy (ACT), an evidence-based approach shown to reduce symptoms across diverse conditions, including anxiety [22,23] chronic pain [13,24], and depression [25,26]. Meta-analytic findings indicate a robust inverse association between PF and psychological distress across populations [27]. Within disability contexts, PF may help disrupt rigid, self-critical thinking patterns that emerge from stigma and shame, as well as mitigate the cognitive fusion that accompanies negative self-evaluation [7,28,29]. By cultivating awareness that thoughts are transient mental events rather than accurate reflections of self or reality, PF supports movement from avoidance and rumination toward acceptance and values-based behavior, thereby enhancing resilience and psychological well-being [19,30]. Empirical evidence further suggests PF mediates the association between stigma-related processes and anxiety and depression among individuals with chronic illness [7] and contributes to reductions in pain-related anxiety, depression, and disability in ACT interventions for chronic pain [20,31]

Self-compassion (SC) may also play a central role in buffering the stigma–shame–distress pathway among people with physical disabilities. Conceptually, SC comprises three interrelated components: self-kindness, recognition that suffering is a universal experience, and mindful awareness [32]. Whereas shame is characterized by self-criticism and activation of the threat protection system, self-compassion is associated with self-kindness and engagement of the soothing-affiliative system [33,34]. Meta-analytic evidence demonstrates consistent associations between SC and enhanced well-being [35] and reduced psychopathological symptoms [36]. A meta-analysis of randomized controlled trials further indicates that SC-based interventions significantly increase self-compassion and reduce depression and anxiety [37]. In disability populations, SC has been negatively associated with stress, depression, anxiety, pain-related outcomes, and disability-related functional impairment, and positively linked to adaptive coping, self-regulation, well-being, and daily functioning [9,38–40].

Although the relationships between depression, shame, PF, and SC have been examined in several studies, the protective impact of PF and SC among people with physical disabilities has yet to be explored. To fill this gap, the present study examined whether PF and SC attenuate the influence of shame on depression among people with physical disabilities. It was hypothesized that:

1. PF and SC would negatively predict depression, over and beyond variance accounted for by shame, and
2. PF and SC would moderate the relationship between shame and depression, such that the relationship between shame and depression would be stronger at low levels of PF/SC and would be weaker at high levels of PF/SC.

## Methods

### Participants and procedure

Following Pacific University IRB approval, participants were recruited through Qualtrics’ online panel service from June 1–15, 2022. Participants completed self-report measures online via Qualtrics and were offered an incentive for their time (a $4 credit toward a gift card). Participants provided written informed consent and were ensured their participation was voluntary and their responses were confidential. Inclusion criteria included living in the U.S., fluency in English, and access to the internet for the survey. Participants under the age of 18 and over the age of 70 were excluded from the study. A total of 207 participants who self-identified as a person with a physical disability consented to participation. An a priori power analysis (G*Power 3.1.9.7. [41]) indicated that a total sample of 129 participants was required to achieve a power of  .95 when employing a *p* < .05 criterion for statistical significance.

The final sample was 44% (*n* = 90) female, 40% (*n* = 83) male, 14% (*n* = 28) nonbinary, and 3% (*n* = 6) transgender and ranged in age from 18-71 years old (*M =* 44.13, *SD =* 17.36). In terms of race and ethnicity, 52% (*n* = 107) of the sample identified as White American, 18% (*n* = 38) as multiracial, 18% (*n* = 38) as Black/African American, 6% (*n* = 13) as other, 4% (*n* = 7) as Asian American, and 2% (*n* = 4) as Native American. Approximately 30% (*n* = 63) of the sample identified as Latino/a. Years of education completed ranged from 6-26 (*M =* 13.74, *SD =* 2.73) and 40% (*n* = 82) were single, 32% (*n* = 66) married, 13% (*n* = 26) divorced, 10% (*n* = 21) widowed, and 6% (*n* = 12) cohabitating.

### Measures

The External and Internal Shame Scale (EISS; [42]) is an 8-item self-report measure that assesses external and internal shame. Items are rated on a 5-point Likert-type scale (0 = *Never* to 4 = *Always*). Total scores are calculated by summing all items, with scores ranging from 0 to 32, and higher scores reflect a greater degree of shame. The EISS has demonstrated acceptable internal consistency in the validation study (α = .89), and excellent reliability (α = .94; *ω =* .94) in the current study.

The Depression, Anxiety, and Stress Scale-21 (DASS-21; [43]), a 21-item version of the DASS [44], is a three-factor measure of cognitive and physical experiences of negative emotional states (depression, anxiety, and stress). Only the depression subscale was used in this study. The depression factor consists of seven items rated on a 4-point Likert-type scale (0 = *did not apply to me at all* to 3 = *applied to me very much or most of the time*). Factor scores are summed and multiplied by two, resulting in scores ranging from 0 to 42, with higher scores indicating greater depression. The DASS-21 depression factor has demonstrated evidence of good to excellent internal consistency (α = .88), and in the current study, it demonstrated excellent reliability (α = .94; *ω =* .94).

The Comprehensive Assessment of Acceptance and Commitment Therapy Processes (CompACT; [45]) is a 23-item self-report measure of psychological flexibility and related processes targeted by ACT. Items on the CompACT are scored on a 7-point Likert scale (0 = *strongly disagree* to 6 = *strongly agree*) ranging from 0 to 161, and higher scores indicate greater psychological flexibility. The CompACT demonstrated excellent internal consistency in the validation (α = .90) and current studies (α = .83; *ω =* .82).

The Self-Compassion Scale-Short Form (SCS-SF; [46]) is a 12-item version of the 26-item SCS [47]). It assesses kindness and understanding toward oneself in instances of pain or failure, perception of one's experiences as part of the larger human experience, and the ability to hold painful thoughts and feelings in mindful awareness. Items are rated on a 5-point Likert-type scale (1 = *almost never* to 5 = *almost always*). The SCS-SF ranges from 12 to 60, with higher scores indicating greater self-compassion. The SCS-SF demonstrated good internal consistency in the validation (α = .87) and current (α = .89; *ω =* .89) studies.

### Data analyses

Statistical analyses were performed using SPSS for Macintosh version 30.0 at an alpha level of 0.05. To test hypothesis 1, a multiple regression was conducted to assess if PF and SC significantly predicted depression, over and beyond variance accounted for by shame. To test hypothesis 2, two multiple regressions were conducted to assess if PF and SC (separately) moderated the impact of shame on depression. More specifically, a cross product was created using the shame and PF and shame and SC products to create the interaction terms. These interaction terms were entered in separate blocks to assess whether they accounted for residual variance above and beyond the predictors in Block 1. All continuous predictor variables were centered prior to conducting the regressions. Significant interactions were probed via simple slope analyses using Hayes’ Process Macro for IBM SPSS v5.0 [48]. There were very little missing data (< 2%) and they were missing completely at random; we therefore used a complete case analysis approach [49].

## Results

All variables approximated a normal distribution, with no univariate or multivariate outliers. Age, gender, relationship status, race, and ethnicity were explored as potential covariates in an initial regression. None were statistically significant (all *p*’s > .20) and therefore no covariates were included in hypothesis testing.

*Hypothesis 1*

Consistent with prediction, PF (β = -.16, *p* = .003, *sr*^2^ = .04) and SC (β = -.25, *p* < .001, *sr*^2^ = .08) were statistically significant predictors of depression, over and beyond variance accounted for by shame (β = .55, *p* < .001, *sr*^2^ = .37).

*Hypothesis 2*

Consistent with prediction, shame (β = .46, *p* < .001, *sr*^2^ = .28), PF (β = -.20, *p* < .001, *sr*^2^ = .04), and their interaction term (β = -.12, *p* < .001, *sr*^2^ = . 02) were statistically significant predictors of depression. Simple slope analyses revealed that shame predicted depression at low (β = .58, *p* < .001) and high (β = .33, *p* < .001) levels of PF; however, the relationship was weaker at high levels of PF. Also consistent with prediction, shame (β = .42, *p* < .001, *sr*^2^ = .18), SC (β = -.24, *p* < .001, *sr*^2^ = .06), and their interaction term (β = -.08, *p* < .001, *sr*^2^ = . 02) were statistically significant predictors of depression. Simple slope analyses revealed that shame predicted depression at low (β = .48, *p* < .001) and high (β = .27, *p* < .001) levels of SC; however, the relationship was weaker at high levels of SC.

## Discussion

The purpose of this study was to determine if PF and SC negatively predict depression and buffer the impact of shame on depression in adults with physical disabilities. Adults with physical disabilities often share a common experience of shame due to the internalization of defectiveness, which can lead to negative self-evaluations commonly associated with depression [7–9]. Despite this, we found that PF and SC predicted depression over and beyond shame, suggesting that they account for aspects of depression not fully explainable by shame. This supports the nascent literature suggesting that PF and SC negatively predict depression in disability populations, including those experiencing chronic pain [13,28]. PF may help people remain open to distressing internal experiences and engage with valued action to avoid entanglement in rumination cycles and respond more adaptively, thereby reducing the risk of depression. Similarly, SC is a practice of being kind and accepting of oneself, which may help reduce negative self-evaluations, such as shame, that lead to depression. These two skills may be particularly relevant for adults with physical disabilities who often experience various forms of discrimination, including “othering”, accessibility barriers, and unpredictable environmental demands [10,11].

Results also indicated that PF and SC buffered the impact of shame on depression for individuals with physical disabilities. This suggests that the link between shame and depression is not uniform, but varies depending on an individual’s PF and SC. This converges with previous studies that found that high levels of experiential avoidance (the inverse of PF) strengthened the link to depression for adults [19,30,50]. Whereas shame is a negative self-evaluation that may contribute to feelings of depression, PF may reduce the fusion people experience to these evaluations, helping reduce the impact of shame on depression for individuals with physical disabilities. Similarly, SC negatively predicts depression in disability populations [9,38–40] and appears to work by allowing individuals to respond to shame-related distress with understanding rather than self-criticism. Although shame describes what people may be feeling, PF and SC modify how people relate to these feelings, buffering the impact of shame on depression.

There are several limitations to address when interpreting the results of this study. First, self-reported measures introduce subjective and social desirability biases that may influence the results. Even though this study identified PF and SC as moderators of the relationship between shame and depression, this is a cross-sectional study with data collected at a single time point, limiting the ability to make causal claims. Second, there may be unmeasured variables (e.g., physical disability type) influencing the relationship between shame and depression. Although this study included individuals with diverse backgrounds, these findings may not be generalizable to individuals without a physical disability and should be interpreted with caution.

Despite these limitations, these results suggest that interventions designed to enhance PF and SC may be helpful in mitigating the impact of shame on depression among adults with physical disabilities. Given people with physical disabilities are 4.6 times more likely to report frequent mental distress than the general population, these preliminary findings support use of treatments such as mindfulness-based interventions (MBIs) and acceptance and commitment therapy (ACT; [21]) among people with disabilities, both of which have been shown to increase PF and SC (e.g., [27,51]). Interventions integrating elements of MBIs and ACT may synergistically impact one’s relationship to shame and other negative emotions that are common among people with physical disabilities. This aligns with process-based models of depression, which view how an individual's relationship to suffering matters more than what they are experiencing. Given the dearth of research in this area, future studies are needed to determine the impact that cultivating these constructs has on changing the relationship between shame and depression over time.

## Supporting information

S1 Checklist STROBE Checklist for Cross-Sectional Studies. (DOCX)

S1 Data Dataset. (XLSX)

S2 Data Data Dictionary. (DOCX)
