# Supplementary material for: Psychological flexibility and self-compassion buffer the impact of shame on depression among adults with physical disabilities
Source: PLOS Ment Health. 2026 Sep 9;3(9):e0000707. doi: 10.1371/journal.pmen.0000707 (PMC13557337; doi:10.1371/journal.pmen.0000707)
Supplement: Supplementary file 2 [file pmen.0000707.s003.docx]

**Data Dictionary**

| Variable | Position | Label |
| --- | --- | --- |
| Age | 1 | Age in years |
| Gender | 2 | Gender |
| Relationship | 3 | Relationship status |
| Race | 4 | Race |
| Ethnicity | 5 | Ethnicity |
| Education | 6 | Years of education completed |
| COMPTOT | 7 | Comprehensive Assessment of Acceptance and Commitment Therapy Processes |
| SCSTOT | 8 | Self-Compassion Scale |
| DASSD | 9 | Depression subscale of the DASS-21 |
| EISSTOT | 10 | External and Internal Shame Scale |

| **Variable Values** | | |
| --- | --- | --- |
| Value | | Label |
| Gender | 1 | Nonbinary |
|  | 2 | Transmale |
|  | 3 | Transfemale |
|  | 4 | Cismale |
|  | 5 | Cisfemale |
| Relationship | 1 | single |
|  | 2 | Cohabitating |
|  | 3 | Widowed |
|  | 4 | Married |
|  | 5 | Divorced |
|  | 6 | Other |
| Race | 1 | Black or African American |
|  | 2 | American Indian or Alaska Native |
|  | 3 | European American or White |
|  | 4 | Asian American |
|  | 5 | Biracial or Multiracial |
|  | 6 | other |
| Ethnicity | 1 | Hispanic or Latino |
|  | 2 | Not Hispanic or Latino |
