# Supplementary material for: Psychological flexibility and self-compassion buffer the impact of shame on depression among adults with physical disabilities
Source: PLOS Ment Health. 2026 Sep 9;3(9):e0000707. doi: 10.1371/journal.pmen.0000707 (PMC13557337; doi:10.1371/journal.pmen.0000707)
Supplement: Supplementary file 3 [file pmen.0000707.s004.docx]

**Response to Reviewers**

*We thank the Editor and reviewers for their very helpful feedback on our manuscript. We addressed the concerns raised and we believe the manuscript is much stronger as a result. Our responses are italicized below, and track changes were used to make revisions in the manuscript text.*

1. Please include in your MS all the information removed for blind review as peer review at PLOS Mental Health is not double-blinded

*We removed all blinding.*

2. Please send a completed 'Competing Interests' statement, including any COIs declared by your co-authors. If you have no competing interests to declare, please state "The authors have declared that no competing interests exist". Otherwise please declare all competing interests beginning with the statement "I have read the journal's policy and the authors of this manuscript have the following competing interests:"

*We uploaded a Competing Interests Statement.*

3. We have noticed that you have uploaded Supporting Information files, but you have not included a list of legends. Please add a full list of legends for your Supporting Information files after the references list.

*Our Supporting Information files were required (STROBE checklist and IRB approval letter) and it isn’t clear to us what list of legends would be needed for these files.*

4. In the online submission form, you indicated that "The dataset in the current study is available from the corresponding author upon request." All PLOS journals now require all data underlying the findings described in their manuscript to be freely available to other researchers.

*We uploaded our data and a data dictionary as Supplemental Files.*

5. Please review your reference list to ensure that it is complete and correct.

*We reviewed our reference list to ensure that it is complete and correct.*

6. Reiterate that gap that the study fills.

*We added this statement at the end of the introduction.*

7. Add some more details regarding your analytical approach.

*We added more details in the Data Analysis section.*
